# Effect of gut microbiota-derived metabolites and extracellular vesicles on neurodegenerative disease in a gut-brain axis chip

**DOI:** 10.1186/s40580-024-00413-w

**Published:** 2024-02-10

**Authors:** Na Yeon Kim, Ho Yeon Lee, Yoon Young Choi, Sung Jun Mo, Soomin Jeon, Jang Ho Ha, Soo Dong Park, Jae-Jung Shim, Jaehwan Lee, Bong Geun Chung

**Affiliations:** 1 Department of Biomedical Engineering, Sogang University, Seoul, Korea; 2 Institute of Integrated Biotechnology, Sogang University, Seoul, Korea; 3 R&BD Center, hy Co., Ltd., Yongin, Korea; 4 Department of Mechanical Engineering, Sogang University, Seoul, Korea; 5 Institute of Smart Biosensor, Sogang University, Seoul, Korea

**Keywords:** Gut-brain axis chip, Human iPSCs, Neural differentiation, Metabolites, Extracellular vesicles, Exosome, Neurodegenerative disease

## Abstract

A new perspective suggests that a dynamic bidirectional communication system, often referred to as the microbiome-gut-brain axis, exists among the gut, its microbiome, and the central nervous system (CNS). This system may influence brain health and various brain-related diseases, especially in the realms of neurodevelopmental and neurodegenerative conditions. However, the exact mechanism is not yet understood. Metabolites or extracellular vesicles derived from microbes in the gut have the capacity to traverse the intestinal epithelial barrier or blood–brain barrier, gaining access to the systemic circulation. This phenomenon can initiate the physiological responses that directly or indirectly impact the CNS and its function. However, reliable and controllable tools are required to demonstrate the causal effects of gut microbial-derived substances on neurogenesis and neurodegenerative diseases. The integration of microfluidics enhances scientific research by providing advanced in vitro engineering models. In this study, we investigated the impact of microbe-derived metabolites and exosomes on neurodevelopment and neurodegenerative disorders using human induced pluripotent stem cells (iPSCs)-derived neurons in a gut-brain axis chip. While strain-specific, our findings indicate that both microbial-derived metabolites and exosomes exert the significant effects on neural growth, maturation, and synaptic plasticity. Therefore, our results suggest that metabolites and exosomes derived from microbes hold promise as potential candidates and strategies for addressing neurodevelopmental and neurodegenerative disorders.

## Introduction

Alterations in the composition of the gut microbiome have been associated with a range of neurodevelopmental [1], neurodegenerative [2, 3], and neuropsychiatric disorders [4]. While there is limited knowledge regarding the mechanisms through which microbes engage with each aspect of the gut-brain axis, a growing body of evidence supports that the intestinal microbiota influences the gut-brain interactions. Recent studies have shown that the gut microbiome plays an important role in regulating neural and glial cell function by interfering with neural and glial cytogenesis and activation through the gut-brain axis, which consists of the enteric environment system and the central nervous system (CNS) [5]. Moreover, within the context of the gut-brain axis, the gut microbes have been observed to intricately regulate neural development and function by modulating the production of various neurotransmitters [6]. Therefore, the correlation between gut microbes and the brain is not solely attributed to the presence of bacteria, but is likely a result of their metabolites or bacteria-derived molecules.

Gut microbes play a pivotal role in transformation and metabolism of dietary and host-derived molecules, giving rise to a diverse array of metabolites with both local and systemic effects [7]. These metabolites traverse the circulation to reach the CNS, thereby establishing bidirectional communication between the cells and gut microbiomes [8, 9]. In particular, within this array of metabolites, neurotransmitters (e.g., serotonin, dopamine, noradrenaline, and gamma-aminobutyric acid (GABA)) undergo indirect regulation through the presence of specific gut bacteria [10]. They can be directly produced by distinct bacterial strains. Neurotransmitters and their precursors, hormone-like metabolites, and short-chain fatty acids (SCFAs) transport or diffuse across the epithelial barrier to the blood, which can potentially influence the CNS [11]. Furthermore, SCFAs generated by gut microbes play a role in regulating the function of peripheral immune cells as well as neural and microglial function [12, 13]. This modulation is crucial for maintaining the homeostasis of brain immunity and managing neuroinflammation [14]. Additionally, the bacterial metabolites regulate signaling and neurotrophic factors including brain-derived neutrotrophic factor (BDNF), nerve growth factor (NGF), and glial cell line-derived neurotrophic factor (GDNF) [15]. These factors can maintain the neural and synaptic growth, survival, and differentiation, while modulating memory and learning functions in neurodegenerative diseases [16, 17]. As a result, alterations in the composition of the gut microbiota can impact the differentiation of neural stem cells (NSCs) and the processes of neurogenesis or neurodegenerative diseases. A number of studies have highlighted the significance of metabolites as crucial signaling molecules generated by bacteria and utilized by the host [18–20]. However, there is limited in vitro data on whether gut-derived bacterial metabolites influence neurodevelopment and neurodegenerative diseases.

Recent studies have unveiled a potential additional method of communication between bacteria and their hosts: extracellular vesicles (EVs). EVs generated by intestinal bacteria have been suggested as a possible significant controller of cross-species and cross-organ communication, serving a crucial role in the normal functioning of the body and the brain's physiological dysfunction [21]. The gut microbe-derived extracellular vesicles (GMEVs) have the capacity to be released into the gut lumen and diffuse into the bloodstream, initiating distinct signaling pathways within the brain via the systemic circulation [22]. These EVs can exert diverse effects on various types of brain cells (e.g., neurons, astrocytes, and microglia) due to the abundant and diverse array of proteins and small ribonucleic acids [23, 24]. Nevertheless, the effect of the GMEVs in gut-brain axis on neural differentiation and neurodegenerative disease is still poorly understood. This lack of understanding is primarily due to the absence of physiological relevance between the living human body and in vitro experiments, given the challenges associated with dynamic environments, adequate perfusion, and experimental reproducibility.

In this study, we aim to recapitulate the physiological environment and functionality of the gut-brain axis chip by precisely mimicking the crucial organotypic cellular architecture, functionality, biochemical factors, and biophysical cues. To address this, we analyzed the effects of the gut chip-mediated metabolites and GMEVs on the neural differentiation of human induced pluripotent stem cells (iPSCs). We also utilized the neurodegenerative model to determine the neuroprotective effects of gut microbiota-derived metabolites and EVs in a gut-brain axis chip.

## Materials and methods

### Fabrication of gut-brain axis chip

The gut and brain chip was designed using Autocad (Autodesk, CA, USA). The gut chip master mold was made by two-step lithography process. SU-8 100 photoresist (MicroChem Corp., MA, USA) was deposited and spin-coated with 2000 rpm for 30 s on a 4-inch silicon wafer and was baked at 65 °C for 20 min and 95 °C for 1 h, respectively. Ultraviolet (UV) light was exposed for 40 s with UV aligner (MDA-400LJ, Midas System Co. Ltd, Daejeon, Korea) through a photomask and unexposed photoresist was developed for 12 min to fabricate the microchannel. The brain chip master mold was made by two-step lithography process as previous reported [25]. A first layer of SU-8 5 (5 μm in thickness) was patterned on a 4 inch silicon wafer to create bridge channels and second layer of SU-8 100 (150 μm in thickness) was patterned to align the first layer to create two main channels. The polydimethylsiloxane (PDMS)-based gut chip mold was prepared using a 10:1 mixture of a silicone elastomer and curing agent (Sylgard 184, Dow Corning Corp., MI, USA). The elastomer mixture was placed in a vacuum desiccator (Lab Companion, Daejeon, Korea) for 30 min to remove air bubbles and was polymerized at 85 °C for 1 h for curing. The polymerized gut chip molds and slide glass were treated with in a plasma cleaner (Femto Science, Korea). For an osmotic pump, PDMS cubic chambers (1 × 1 × 1 cm) with one cellulose membrane face were fabricated to make the osmotic pump using conventional protocols as previously described [26]. The adhesion between the PDMS chamber and the cellulose membrane was adhered using the PDMS solution as an adhesive. In preliminary experiments, the osmotic experiments were conducted to evaluate the pumping ability of the osmotic pump [25]. The deionized water was used as a buffer solution and polyethylene glycol (PEG) (Sigma-Aldrich, MO, USA; 2000 molecular weight) solution was used as a driving agent.

### Preparation and Caco-2 cells and bacterial seeding in a gut-brain axis chip

The gut chip was coated overnight with 0.1 mg/mL poly-D-lysine (Thermo Fisher Scientific, MA, USA). Human intestinal epithelial cells (Caco-2) (ATCC clone HTB-37) were cultured in Minimum Essential Medium (Thermo Fisher Scientific, MA, USA), supplemented with 10% fetal bovine serum (Thermo Fisher Scientific, MA, USA), 100 U/mL penicillin, 100 µg/mL streptomycin (Thermo Fisher Scientific, MA, USA), and L-glutamine (Thermo Fisher Scientific, MA, USA). Caco-2 cells (20 μL, 2 × 10^7^ cells/mL) were seeded into the microchannel and were incubated overnight. After adherence to the microchannel, the non-adherent cells were removed. The outlet of the chip was then connected to an osmotic pump. Lactobacillus casei Hy2782 (Hy2782) and Lactobacillus plantarum Hy7714 (Hy7714) probiotics, obtained from hy Co., Ltd. (Yongin-si, Korea), were cultured in Man, Rogosa, and Sharp (MRS) broth (BD, Franklin Lakes, NJ, USA) at 37 °C for 24 h. For live bacteria analysis on the chip, probiotics were stained using SYTO® 9 from the LIVE/DEAD^®^ Bac-Light™ Bacterial Viability Kit (Thermo Fisher Scientific, MA, USA). Subsequently, the bacterial cells were harvested by centrifugation (3500 rpm, 15 min), washed three times with phosphate-buffered saline (PBS), and resuspended in the cell culture medium at a concentration of 10^6^ CFU/mL. The bacterial cells were then loaded into the microchannel and the chip was connected to the osmotic pump. The bacterial cells were filtered using a 0.22 μm syringe filter (Sartorius, Göttingen, Germany).

### Human iPSCs culture

The human iPSC WTC cells (passage 50 to 60) were cultured in 6-well plates coated with 1% Geltrex (Thermo Fisher Scientific, MA, USA) using mTeSR^TM^1 medium (Stem Cell Technologies, Vancouver, Canada) with daily medium replacement. Passage was conducted by incubating cells with ReLeSR ^®^ reagent (Stem Cell Technologies, Vancouver, Canada) for 4 min at room temperature, following wash with PBS solution, when cells reached 80–90% confluency. Cells were collected by pipetting with culture medium and plated at a 1:3 ratio on new Geltrex-coated 6-well plates, allowing them to grow to 90–100% confluence. Cells were cultured with mTeSR^TM^1 medium (Stem Cell Technologies, Vancouver, Canada) supplemented with 10 µM Rho-associated kinase (ROCK) inhibitor Y-27632 (Tocris Bioscience, Bristol, UK) to enhance cell survival. After 24 h, the medium was replaced with mTeSR^TM^1 medium without the ROCK inhibitor.

### Commitment of human iPSCs into NSCs (iNSCs)

The differentiation of NSCs from human iPSCs was followed by previous protocols [27]. To form three-dimensional (3D) spheres, human iPSCs were dissociated with ReLeSRTM (Stem Cell Technologies, Vancouver, Canada) and placed on non-adherent plates to facilitate cell aggregation. On Day 1, the cells were seeded as clumps (80–100 cells/clump) in mTeSR^TM^1 medium (Stem Cell Technologies, Vancouver, Canada). The mTeSeR1 (Stem Cell Technologies, Vancouver, Canada) medium was changed daily until Day 8. On Day 9, the floating spheres were transferred to tissue culture plates with Dulbecco’s modified Eagle’s medium (DMEM)/F12 (Life Technologies, CA, USA) supplemented with ITS (Stem Cell Technologies, Vancouver, Canada) and were cultured for 10 days with an additional 20 ng/mL of fibronectin for attachment and growth. On day 18, the cell clones from one well of a 6-well plate were mechanically scraped into floating fragments. Subsequently, these fragments were plated on a surface coated with 50 μg/mL poly-L-ornithine (Sigma Aldrich, MO, USA) and 0.5 mg/mL laminin (Sigma Aldrich, MO, USA) to facilitate attachment and outgrowth. The maintenance medium comprised DMEM/F12 (Invitrogen, MA, USA), supplemented with 1% N2 (Life Technologies, MA, USA), 2% B27 medium (Life Technologies, MA, USA), 20 ng/mL basic fibroblast growth factor (bFGF, R&D System, MN, USA), and 20 ng/mL epidermal growth factor (EGF, Invitrogen, MA, USA).

### Human iNSC differentiation in a brain chip

**Fig. 1:**
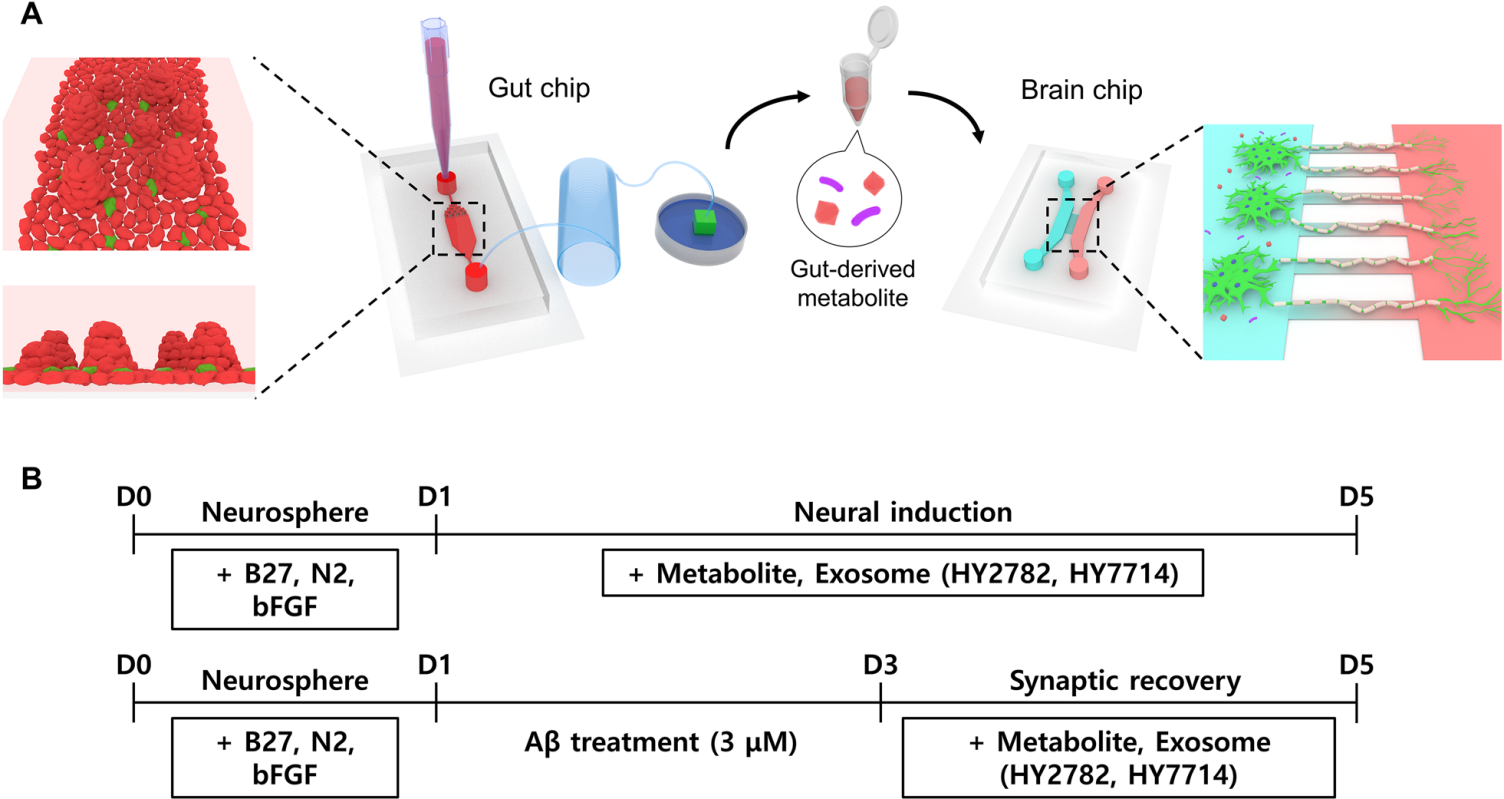
**A** Schematic of the experimental setup for a gut-brain axis chip system. The gut chip used human epithelial Caco-2 cells to establish an intestinal lumen, while the brain chip with bridge microchannels guided the axonal growth of neurons. **B** The upper scheme illustrates the differentiation of neural cells from human iNSCs, while the lower scheme depicts the induction of an Alzheimer's disease model

Before seeding to the brain chip, the chips were coated with 50 μg/mL poly-L-ornithine (Sigma Aldrich, MO, USA) and 0.5 mg/mL laminin (Sigma Aldrich, MO, USA). Human iNSCs were seeded into the left channel and neurospheres (10 neurospheres/compartment) were loaded into the right channel of the compartment. After loading to the brain chip, the culture medium was replaced with neural basal medium, comprising DMEM/F12 (Invitrogen, MA, USA) supplemented with 1% N2 (Life Technologies, MA, USA) and 2% B27 medium (Life Technologies, MA, USA). Subsequently, human iNSCs were treated with metabolites or exosomes derived from Hy2782 and Hy7714 to culture for 5 days. The neural differentiation protocol is shown in Fig. 1B.

### Immunofluorescence and imaging analysis

The cells grown in the gut chip and human iNSCs cultured in the brain chip were immunostained by following procedure. The cells were fixed in 4% paraformaldehyde (Sigma Aldrich, MO, USA) for 30 min. After washing with PBS, the cells were permeabilized with 0.1% Triton X-100 (Samchun, Korea) in PBS for 30 min at room temperature. To reduce non-specific binding, the cells were treated with 3% bovine serum albumin (BSA, Sigma Aldrich, MO, USA) in PBS for 1 h at room temperature. Each sample was incubated with its respective antibodies. The antibodies included Alexa Fluor 594 Phalloidin (1:200, Invitrogen, MA, USA), Anti-ZO-1 (1:200, Invitrogen, MA, USA), Anti-Villin (1:200, Invitrogen, MA, USA), Anti-Tuj1 (1:1000, Biolegend, CA, USA), Anti-Neurofilament (1:200, Sigma Aldrich, MO, USA), Anti-MAP2 (1: 200, Abcam, Cambridge, UK), Anti-PSD95 (1:200, Abcam, Cambridge, UK), Anti-GAP43 (1: 200, Abcam, Cambridge, UK), Anti-NESTIN (1:200, Abcam, Cambridge, UK), and Anti-NeuroD1 (1:200, Cell Signaling Technology, Cambridge, UK). These antibodies were diluted with PBS and the cells were incubated overnight at 4 °C. After washing with PBS, the samples were incubated with the secondary antibodies: Alexa Fluor 488 donkey anti-mouse IgG (1:200, Invitrogen, MA, USA), Alexa Fluor 488 donkey anti-rabbit IgG (1:200, Invitrogen, MA, USA), Alexa Fluor 594 donkey anti-mouse IgG (1:200, Invitrogen, MA, USA), and Alexa Fluor 594 donkey anti-rabbit IgG (1:200, Invitrogen, MA, USA) overnight at 4 °C. All samples were counter-stained with 4′,6-diamidino-2-phenylindole (DAPI, 1 mg/mL diluted in staining solution, Sigma Aldrich, MO, USA) for 10 min at room temperature. Immunostained images were captured using an inverted confocal laser scanning microscope (LSM710, Carl Zeiss, Jena, Germany). Image J software was employed for the analysis of the confocal fluorescence images.

### Statistical analysis

All experiments were conducted in triplicate or more to ensure the reproducibility and reliability of the results. The results are presented as mean ± standard deviation or standard error of the mean. For statistical analyses, the mann–whitney U test or one-way analysis of variance (ANOVA) with Bonferroni’s multiple comparisons test was performed using GraphPad Prism version 8.0 (GraphPad Software Inc., CA, USA). Statistically significant differences between groups were denoted as *p < 0.05, **p < 0.01, ***p < 0.001, and ****p < 0.0001. Additionally, the black line above the histogram represents the comparison between the two groups.

## Results and discussion

### Development of a gut-brain axis chip

To explore the interaction of gut-derived metabolites within a human brain microenvironment, we developed a gut-brain axis chip. The experimental schematic in Fig. 1A illustrates the design of the gut chip, featuring a single large channel (20 mm length, 6 mm width, and 100 μm height) explicitly tailored for Caco-2 cell culture. Micropillars within the channel ensure the uniform distribution of cells, and an osmotic pump directs the intestinal flow towards the outlet. The brain chip was also manufactured using a two-step photolithography process to consist of two main channels and bridge channels. The main channels (16 mm length, 1 mm width, and 150 μm height) comprised a left channel for the culture of human iNSCs and a right channel for the derivation of NSC axons. Bridge channels (500 μm length, 10 μm width, and 5 μm height) are strategically designed to distinguish axonal and dendritic compartments from the NSC culture channel. Interconnections between the cell culture channel and the derivative channel are facilitated by bridge channels that can act as a fluidic resistance for axonal guidance and isolation.

### Intestinal cell differentiation in a gut chip

**Fig. 2:**
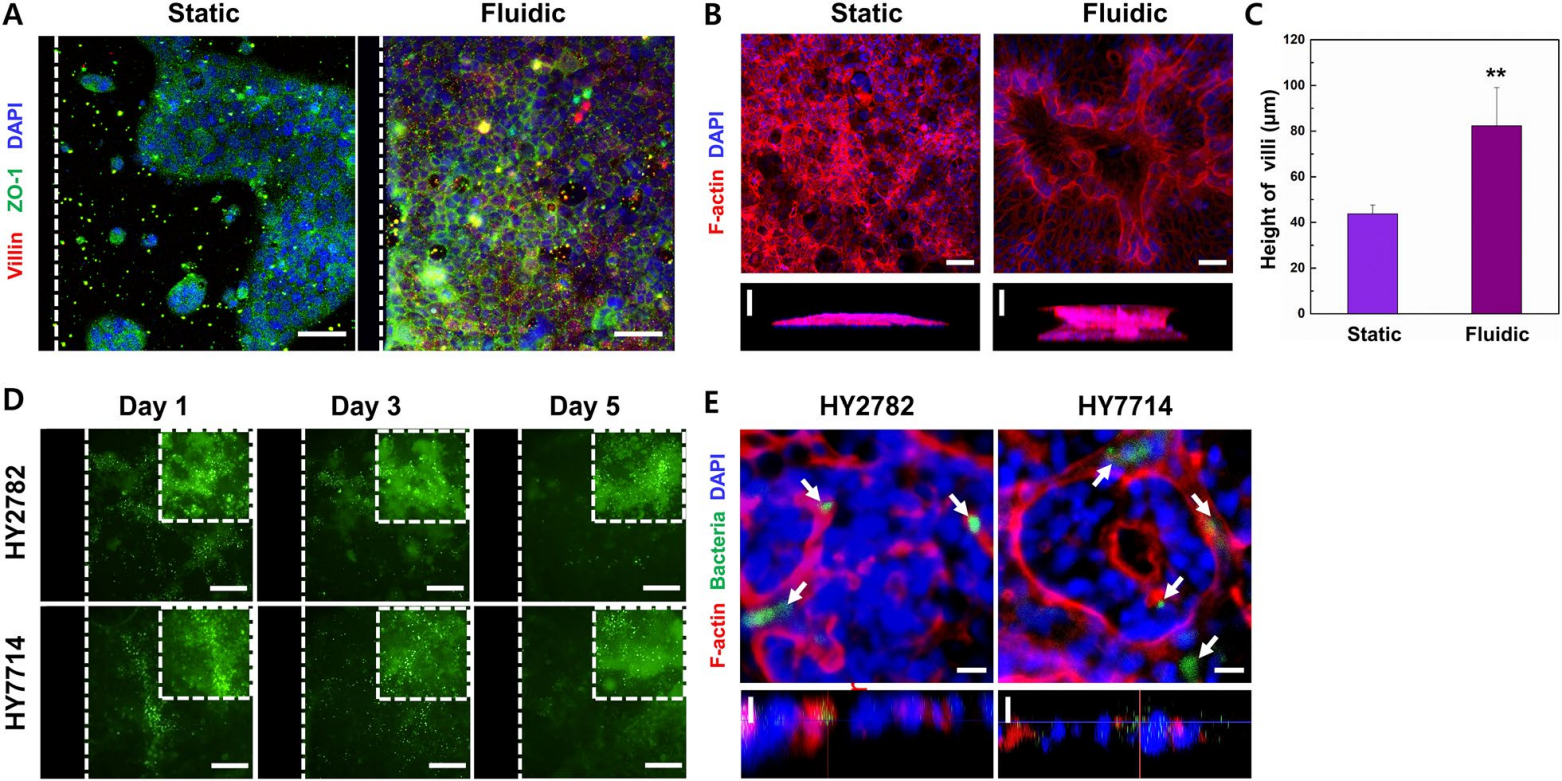
Morphology of Caco-2 epithelial cells cultured in the microfluidic-based gut chip for 5 days. **A** Distribution of the tight junction protein, ZO-1, and villin in Caco-2 cell monolayers. White dot lines indicate the microchannel wall. Scale bars are 100 μm. **B** Morphological analysis of polarized columnar epithelium. Fluorescence confocal micrographs (vertical cross-sectional views at 5 days after onset) highlight cell shape and polarity. Horizontal scale bars are 50 μm and vertical scale bars are 25 μm. **C** Average height of Caco-2 cells grown in the gut chip. **D** Microbial co-culture on a human intestinal epithelial layer in the gut chip. White dot lines indicate the microchannel wall. Scale bars are 100 μm. **E** Fluorescence confocal micrographs depicting colonies of the green-labeled microbiome cultured on the intestinal epithelium on-chip for 5 days. Horizontal scale bars are 50 μm and vertical scale bars are 25 μm

Preceding the investigation into the impact of microbiota metabolites and EVs on neurogenesis and neurodegenerative diseases, we conducted a comparative assessment of Caco-2 cell differentiation under static and fluidic conditions. The growth and differentiation profiles of Caco-2 cells were systematically analyzed under the following conditions: perfusion of cell culture medium through microchannels at a pre-optimized flow rate over 5 days culture period [25]. Immunofluorescence staining confirmed the presence of markers indicative of cellular integrity, polarization, and morphological characteristics under both static and fluidic conditions including ZO-1, villin, and F-actin (Fig. 2A, B). Under the fluidic conditions, smaller and more uniformly sized cell junctions were observed, consistent with the characteristic appearance of polarized cells distinguished by reduced diameter and elongated morphology. Confocal images further demonstrated the presence of villi in a fluidic culture condition, confirming brush border formation and 3D organization, while its absence was evident in static conditions. The Z-stack projection also reveals a undulating profile reminiscent of in vivo intestinal cell morphology. The mean heights of villi produced under static and flow conditions were 43.77 μm and 82.32 μm, respectively, representing an approximately twofold increase under the flow culture condition (Fig. 2C). These results demonstrate that the robust differentiation and polarization of Caco-2 cells cultured on the gut chip that can closely mimic the structure of the intestine in vivo.

Subsequently, the microbiota were introduced to the gut chip to assess the viability and colonization of specific bacterial strains (Fig. 2D, E). Notably, when Hy7714 and Hy2782 were cultured under fluidic conditions, the number of colonized bacteria on the gut chip remained stable, exhibiting no signs of overgrowth even after culturing for 5 days. Confocal images, presented in Z-stack projections, illustrated the presence of colonized bacteria interspersed among the microvilli (white arrow). Prior research has indicated that bacterial overgrowth can rapidly compromise epithelial integrity, emphasizing the importance of inhibiting such overgrowth while preserving epithelial differentiation for the establishment of a stable symbiotic relationship, as observed at in vivo intestinal context [28, 29]. Therefore, the continuous dynamic culture conditions appear to be effective preventing the bacterial overgrowth while sustaining bacterial viability.

### Effect of microbe-derived metabolites on neural differentiation

**Fig. 3:**
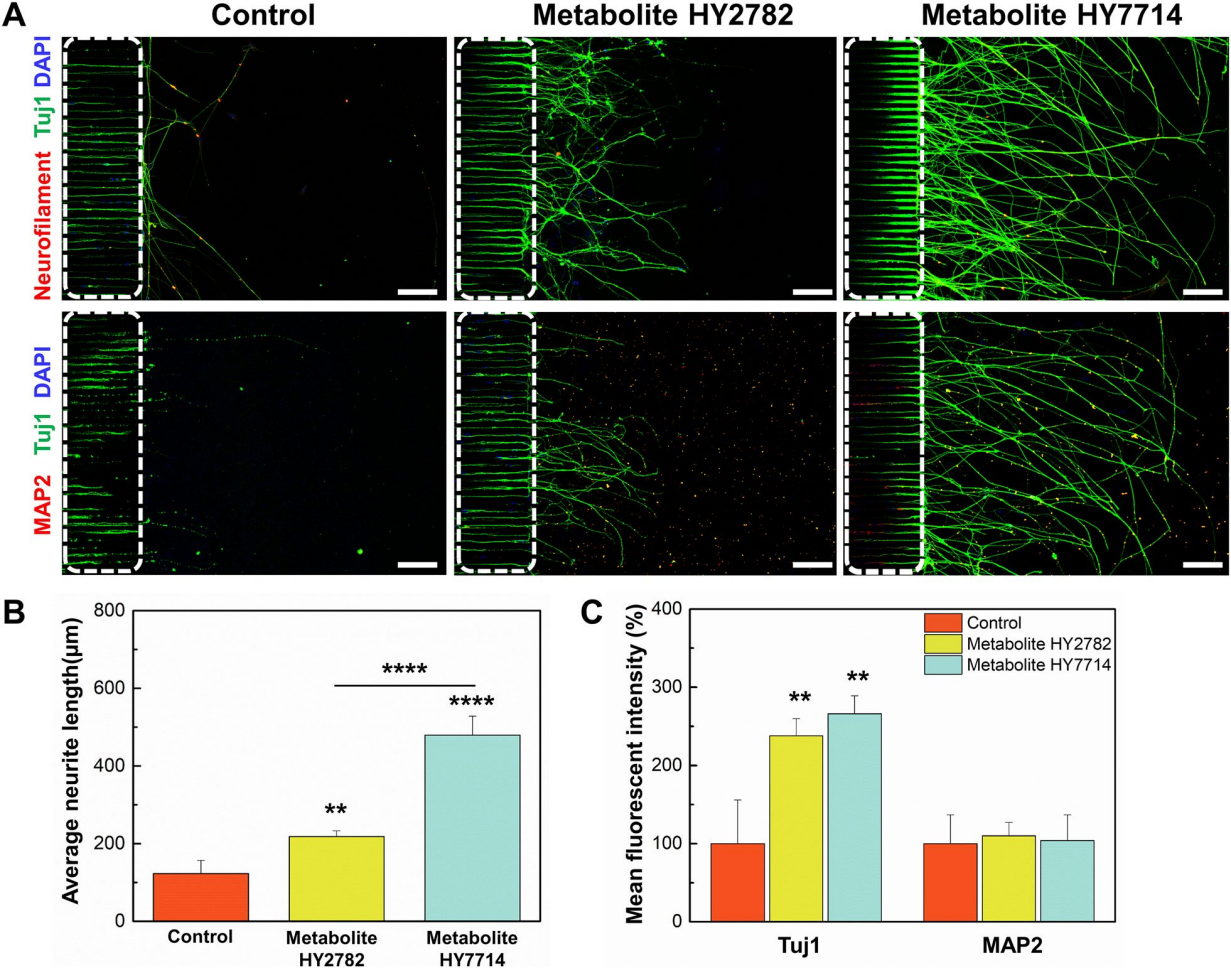
Effect of microbe-derived metabolites on neural growth. **A** Immunofluorescence images of human iNSCs cultured in different conditions in a brain chip for 5 days. White dot lines indicate the bridge microchannels. Scale bars are 100 μm. **B** Analysis of average neurite length in a brain chip after Hy2782- and Hy7714-derived metabolites. **C** Analysis of Tuj1- and MAP2-positive cell intensity in a brain chip after Hy2782- and Hy7714-derived metabolites

Although a number of studies have demonstrated in vivo effects of microbe-derived metabolites on neurogenesis or neurodegenerative diseases, in vitro studies have been limited. Therefore, we utilized the brain chip to observe and quantify whether microbe-derived metabolites affected neural differentiation in vitro. Microbe-derived metabolites were selected through previous screening (data not shown). Neural progenitor cells were seeded in the brain chip and were cultured with medium containing microbe-derived metabolites for 5 days. Immunofluorescence data revealed a significant increase in neurite length in the metabolite treatment group as compared to the control group (Fig. 3). The neurite lengths of the control and metabolite-treated groups were 123 ± 34 μm, 218 ± 15 μm, and 479 ± 61 μm, respectively, with the longest neurite growth observed in the group treated with metabolites derived from Hy7714 (Fig. 3B). Consistent with the results for neurite length, the quantification of the immunofluorescence staining showed high expression of Tuj1, while no statistical difference was observed in MAP2 expression in the metabolite-treated groups (Fig. 3C). The expression of Tuj1 significantly increased by 2.2-fold and 2.5-fold in the metabolite-treated groups derived from Hy2782 and Hy7714, respectively, as compared to the control group (*p* = 0.0041,* p* = 0.0013, respectively). Tuj1 expression becomes prominent as neural progenitor cells commit to a neural fate, indicating the initiation of neurite outgrowth [30]. A recent study has showed that some specific metabolites have great potential in neural health [31]. Interestingly, our result revealed that treatment with microbe-derived metabolites markedly enhanced the neurite outgrowth of iNSCs as compared to the control group. Additionally, we confirm that iNSCs exposed to metabolites derived from gut microbes undergo terminal differentiation, maturing into neurons with more branched neurites. Furthermore, the presence of MAP2 in dendrites is associated with the structural complexity and maturation of neurons [32]. Thus, the low expression levels of MAP2 suggest that cells are in an early neural cell state and have not yet developed the dendritic arbor characteristic of mature neurons. In the context of neural cell differentiation, these findings strongly suggest that microbe-derived metabolites play a crucial role in promoting the growth of neurites during the early development of neurons.

### Microbe-derived metabolites in expression of synapse-related proteins and neural maturation

**Fig. 4:**
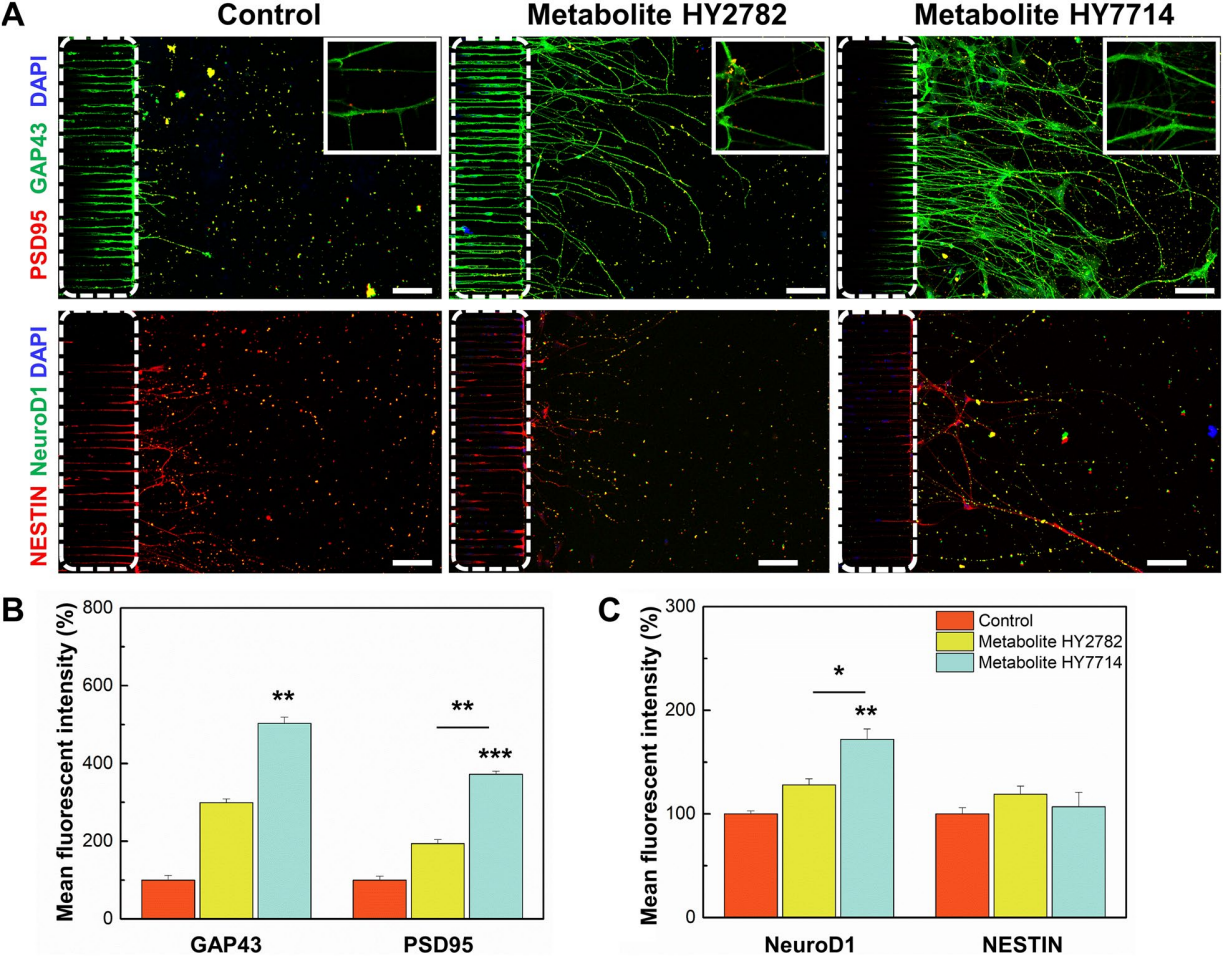
Effect of microbe-derived metabolites on synaptic plasticity. **A** Representative immunofluorescent images of synaptic plasticity-related proteins. Dendrite segments (insets) are enlarged to show individual puncta. White dot lines indicate the bridge microchannels. Scale bars are 100 μm. **B** Analysis of GAP43 and PSD95-positive cell intensity in the brain chip after treatment with Hy2782- and Hy7714-derived metabolites. **C** Analysis of NeuroD1- and Nestin-positive cell intensity in the brain chip after treatment with Hy2782- and Hy7714-derived metabolites

The aforementioned studies have shown that microbial-derived metabolites affect the growth and differentiation of neurons. In this case, we hypothesized that microbial-derived metabolites would also influence the maturation of neural cells. Hence, we analyzed markers associated with synaptogenesis and synaptic plasticity. Post-synaptic density protein-95 (PSD95) plays a crucial role in synaptic plasticity, ensuring the long-term stabilization of synaptic changes [33], while growth associated protein-43 (GAP43) is involved in guiding neural pathways and promoting the branching of neurons [34]. Moreover, GAP43 actively contributes to presynaptic membrane alterations, initiating neurotransmitter release, endocytosis, and recycling of synaptic vesicles, making it a pivotal factor in evaluating neural connectivity. The fluorescence of the synaptic markers (e.g., PSD95 and GAP43) was observed in the groups treated with microbe-derived metabolites (Fig. 4). In particular, the anti-PSD95 staining exhibited bright red fluorescence, localized in proximity to the axons of differentiated neurons. Meanwhile, GAP43-positive green fluorescence was observed in the cell bodies and axons of human iNSC-derived neurons. In Fig. 4B, the expression of PSD95 increased by 1.9-fold and 3.7-fold in Hy2782 and Hy7714-derived metabolite treated group, respectively, as compared to the control. Similarly, the expression of GAP43 increased by threefold and fivefold in the groups treated with metabolites derived from Hy2782 and Hy7714, respectively, with the significantly highest GAP43 expression observed in the Hy7714-derived metabolite-treated group (*p* = 0.0005) (Fig. 4B). Immunofluorescence analysis revealed an increased protein expression of PSD95 and GAP43 in differentiated neurons following exposure to microbe-derived metabolites, suggesting that these metabolites may promote the expression of synaptic proteins. Furthermore, this implies that microbe-derived metabolites may also regulate proteins, such as GAP43 and PSD95, in synaptic plasticity mechanisms. Newborn neurons undergo dynamic neurite remodeling to integrate into existing circuits and form new synapses to contribute to hippocampal function [35, 36]. In the present study, we observed high density of synaptic-related markers in vitro, indicating that the treatment with gut microbe-derived metabolites enhances synaptogenesis in neurogenesis. To assess the differentiation of NSCs into neurons, we compared the expression of the neural differentiation factor 1 (NeuroD1) [37] and neuro-ectodermal marker, nestin [38]. We confirmed a significant increase in the expression of NeuroD1 in group treated with Hy7714-derived metabolites (*p* = 0.0018) (Fig. 4C). However, nestin expression remains low with no significant differences among the groups. Therefore, we determined that gut microbial-derived metabolites, specifically Hy7714, promoted the development of neurons rather than the maintenance of neural progenitors by supporting the expression of neural transcription factors. Collectively, these findings suggest a crucial role for microbe-derived metabolites in fostering the differentiation of neural progenitor cells into mature and functional neurons, thereby making a significant contribution to the establishment of neural structure in the developing nervous system.

### Effect of microbe-derived exosome on neural differentiation

**Fig. 5:**
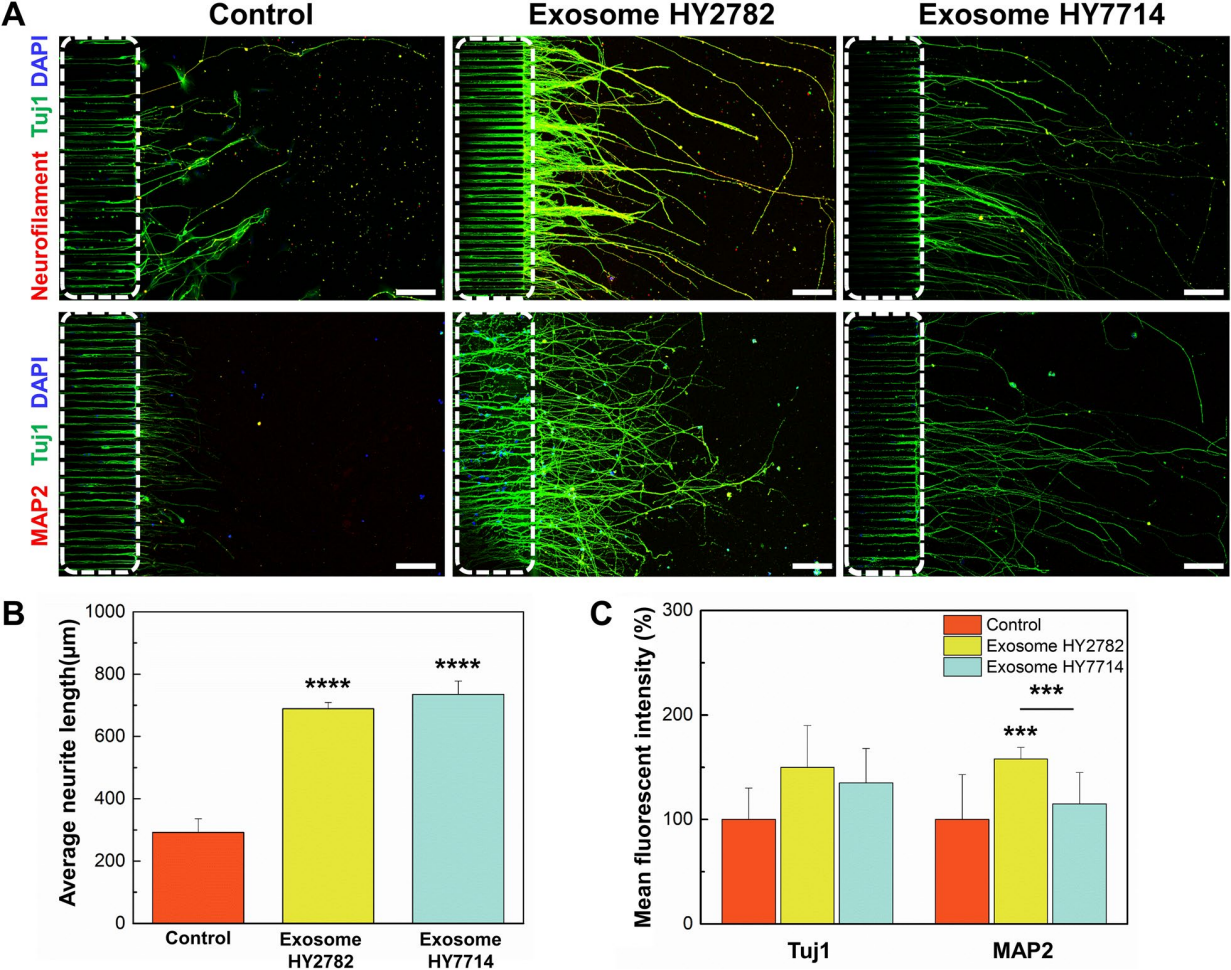
Effect of microbe-derived exosomes on neural growth. **A** Immunofluorescence images of human iNSCs cultured in different conditions in a brain chip for 5 days. White dot lines indicate the bridge microchannels. Scale bars are 100 μm. **B** Analysis of average neurite length in the brain chip after treatment with Hy2782- and Hy7714-derived exosomes. **C** Analysis of Tuj1- and MAP2-positive cell intensity in the brain chip after treatment with Hy2782- and Hy7714-derived exosomes

The impact of microbe-derived exosomes on neural differentiation has not been thoroughly investigated. We conducted co-cultures on brain chips using culture medium supplemented with each exosome (3 × 10^10^ particles/mL). This endeavor aimed to assess the efficacy of NSC differentiation, focusing on selected Hy2782- and Hy7714-derived exosomes based on the outcomes of our previous screening study (data not shown). To quantify and compare the expression levels of various neural markers, the immunofluorescence was performed after 5 days of neural culture. Consistent with the immunofluorescence analysis, we observed the high expression of neural markers, including neurofilament, Tuj1, and MAP2, in the exosome-treated groups. In particular, significantly increased neural length growth was notably observed in the group treated with microbial-derived exosomes than control group (Fig. 5A). The neurite length growth in the control, Hy2782, and Hy7714 exosome treatment groups was 290 ± 44 μm, 689 ± 16 μm, and 735 ± 43 μm, respectively (compared to the control group, *p* < 0.0001 in both group, respectively) (Fig. 5B). Furthermore, we compared the efficacy of neural differentiation in the experimental and control groups (Fig. 5C). The expression of Tuj1, an indicator of neural differentiation development, was increased in both Hy2782 and Hy7714 exosome-treated groups as compared to the control group. To evaluate the neural stabilization and dendrite development, we examined the expression of MAP2, a marker indicative of mature neurons. The findings indicated a significant elevation in MAP2 expression in the group treated with Hy2782-derived exosomes as compared to the control (*p* = 0.0001). Furthermore, upon comparing the groups subjected to exosome treatments, a significant increase in MAP2 expression was observed in the Hy2782-derived exosome-treated group (*p* = 0.0007). These results suggest that exosomes derived from microbes promote the maturation of NSCs into neurons. Based on these results, we determined that microbe-derived exosomes could positively affect not only axon length growth but also dendrite maturation simultaneously.

### Effect of microbe-derived exosomes on expression of synapse-related proteins and neural maturation

**Fig. 6:**
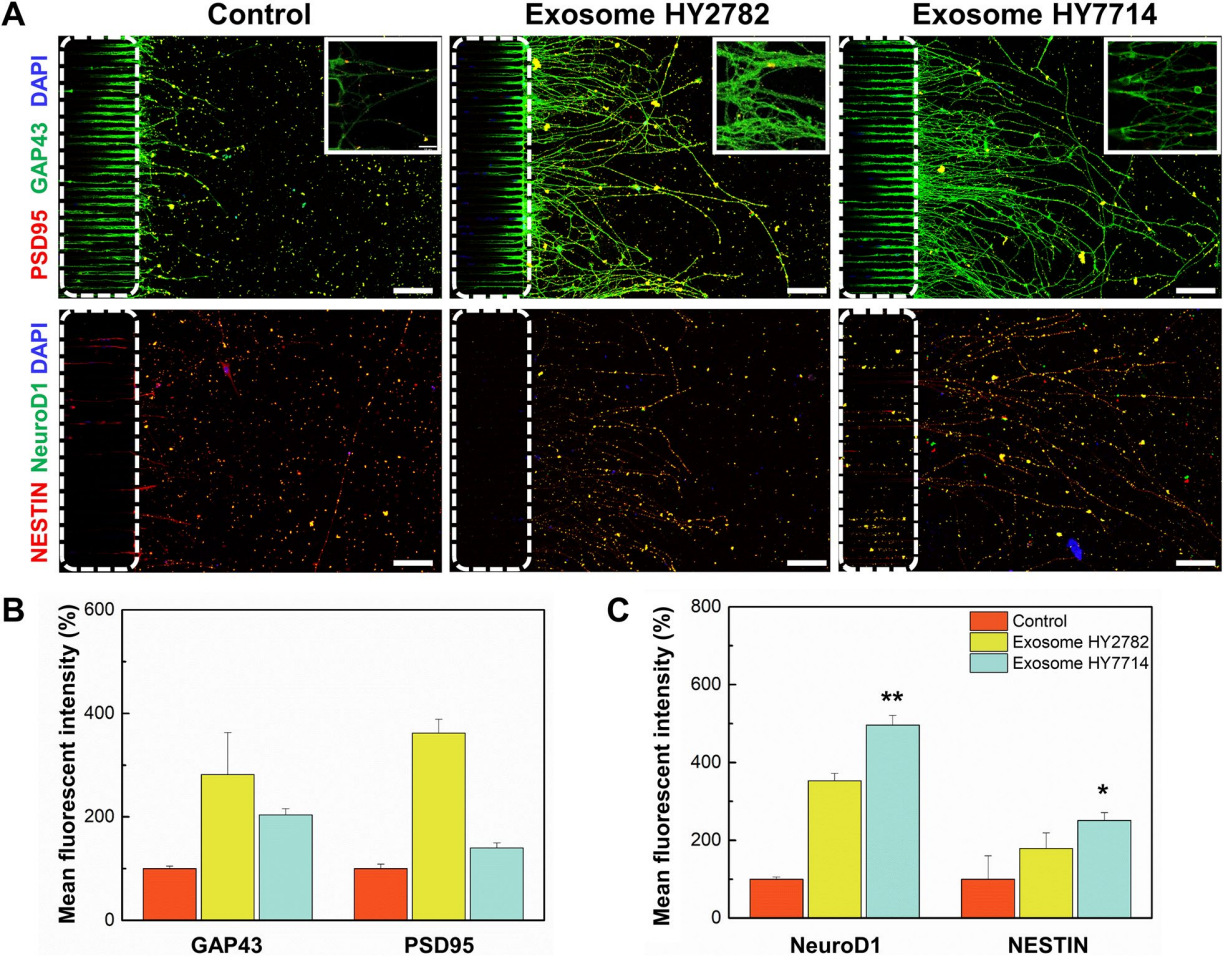
Effect of microbe-derived exosomes on synaptic plasticity. **A** Representative immunofluorescent images of synaptic plasticity-related proteins. Dendrite segments (insets) are enlarged to show individual puncta. White dot lines indicate the bridge microchannels. Scale bars are 100 μm. **B** Analysis of GAP43- and PSD95-positive cell intensity in the brain chip after treatment with Hy2782- and Hy7714-derived exosomes. **C** Analysis of NeuroD1- and Nestin-positive cell intensity in the brain chip after treatment with Hy2782- and Hy7714-derived exosomes

Synaptogenesis plays a critical role in the formation and function of neural network. We examined the synaptogenesis in the developing neural network through the immunofluorescence analysis (Fig. 6, upper panel). Immunofluorescence experiments confirmed an elevation in the expression of synaptogenesis-related markers as compared to the control group (Fig. 6B). In the control group, GAP43 expressions remained at a low level, and identifying the granular immunofluorescence patterns along the outgrowing neurites was challenging. In contrast, GAP43 immunoreactivities highly increased as neurites extended and established connections with neighboring neurons in the exosome-treated groups. The quantification of immunofluorescence data revealed a significant increase in GAP43 levels, particularly in the Hy2782-derived exosome-treated group. The GAP43 levels in this group were 2.8-fold higher than the control group and 1.3-fold higher than Hy7714 exosome-treated group. Additionally, PSD95 exhibited weak expression in the control group. However, within the Hy2782-derived exosome-treated group, PSD95 expression showed a remarkable 3.6-fold increase as compared to the control group and appeared punctate. This observation suggests that Hy2782-derived exosome treatment promoted synapse maturation as compared to both the control and Hy7714 groups.

Both GAP43 and PSD95 play crucial roles in neural network function and maturation. Elevated expression of GAP43 is correlated with enhanced neurite outgrowth and can contribute to the formation of new synaptic connections during neural development [39, 40]. On the other hand, PSD95 is a synaptic protein primarily associated with mature neurons and the postsynaptic density of excitatory synapses [41, 42]. It plays an important role in synapse maturation, synaptic plasticity, and the overall functioning of established synaptic connections. A number of researchers demonstrated that these proteins contributed to different aspects of neural network development [43, 44]. Therefore, we conclude that the processing of microbe-derived exosomes fosters growth and structural changes during the early stages of neurogenesis, organizes and stabilizes synapses, and induces the generation of mature neurons. NeuroD1 is predominantly expressed in the nervous system late in development and is therefore more likely to be involved in terminal differentiation, neural maturation and survival [37, 45]. Additionally, nestin, a pan-neuronal marker, has widely expressed in all neural progenitor cells [46]. There was a significant increase in NeuroD1 expression in the Hy7714-derived exosome-treated group (*p* = 0.0054), consistent with the findings in the Hy7714-derived metabolite-treated group (Fig. 6C). Furthermore, the expression of nestin significantly increased only in the group treated with Hy7714-derived exosomes (*p* = 0.01). In this study, we concluded that even with the treatment of microbe-derived exosomes, the neural cells are still in the early or intermediate stages of development. Nevertheless, it is clear that even at these early developmental stages, the treatment with microbial-derived exosomes has a significant impact on the maturation and differentiation promotion of iNSCs.

### Effect of microbe-derived metabolites and exosome on Alzheimer’s disease model

**Fig. 7:**
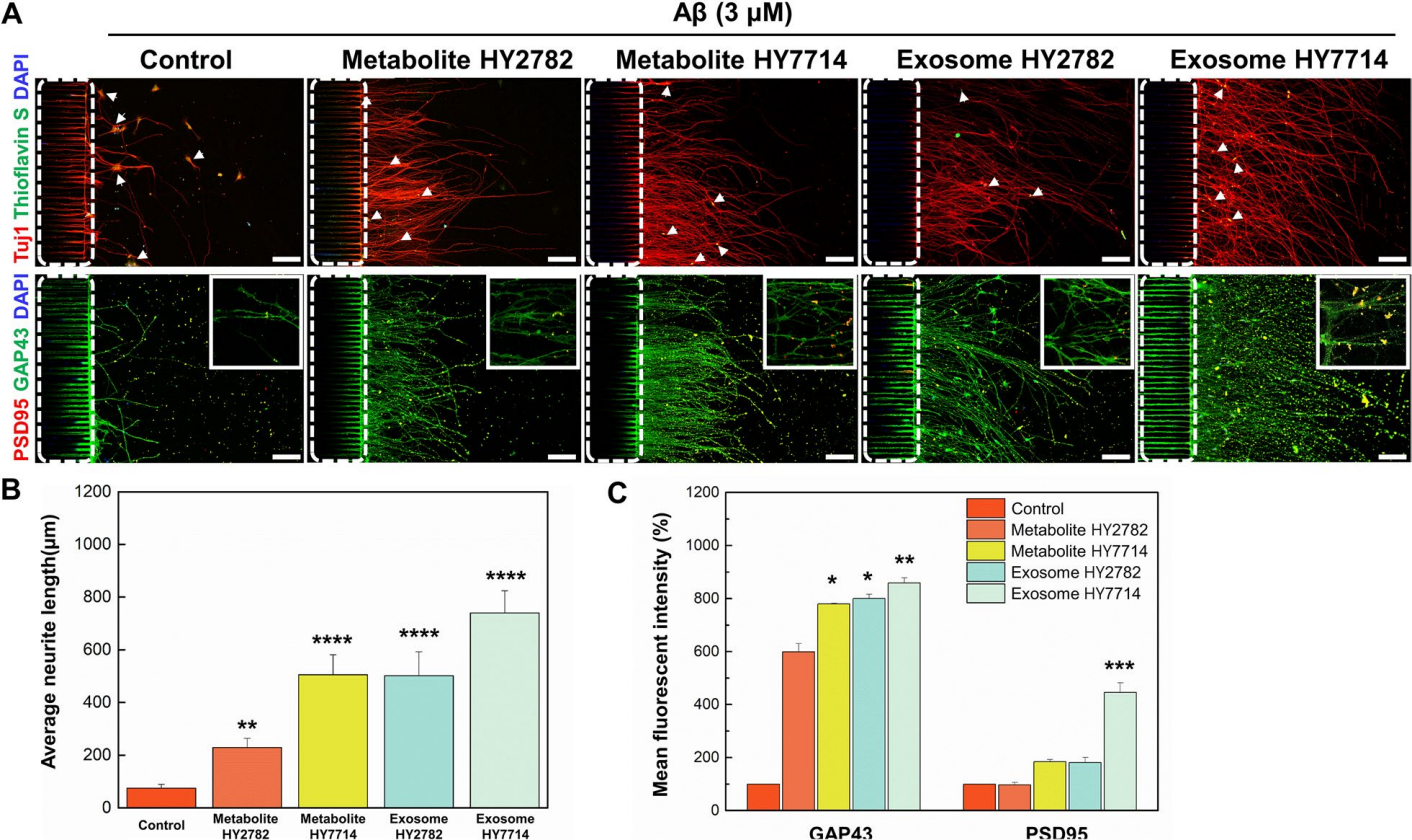
Effect of microbe-derived metabolites and exosomes on in vitro Alzheimer’s disease model. **A** Immunofluorescence images of neurons induced with Alzheimer’s disease model using amyloid, cultured for 5 days under different treatments. White dot lines indicate the bridge microchannels. Scale bars are 100 μm. **B** Analysis of average neurite length in brain chip after treatment with Hy2782- and Hy7714-derived metabolites and exosomes. **C** Analysis of GAP43 and PSD95-positive cell intensity after treatment with Hy2782- and Hy7714-derived metabolites and exosomes

We observed diverse effects of microbial-derived metabolites and exosomes on neural development, growth, and maturation. Considering these microbe-derived metabolites and exosomes as potential therapeutic candidates for disease models, we subsequently established an Alzheimer ‘s disease model to evaluate their effectiveness. The widely acknowledged hypothesis is the amyloid cascade hypothesis [47], with subsequent notable hypotheses including the cholinergic hypothesis [48], tau hypothesis [49], and the relatively recent neuroinflammation hypothesis [50]. Among these hypotheses, we applied the amyloid cascade hypothesis to induce the Alzheimer's disease model. The Fig. 1B illustrates the list of neural differentiation inducers and the schedule of Alzheimer's disease induction experiments. To assess the presence and distribution of amyloid-β in neurons, we stained neurons with thioflavin S, which could bind β-sheet-rich structures to stain amyloid-β. As shown in Fig. 7, the control group displayed an elevation in thioflavin S (amyloid-β plaque indicated white arrow) fluorescence intensity and a reduction in axon growth following amyloid β treatment. In contrast, the metabolite- and exosome-treated groups had significantly more axonal growth and formation of neural network than the control group, despite the amyloid beta treatment (Fig. 7B). In addition, the expression of GAP43, associated with the promotion of axonal growth, significantly increased in groups treated with Hy7714-derived metabolites (*p* = 0.014), Hy2782-derived exosomes (*p* = 0.011), and Hy7714-derived exosomes (*p* = 0.006) as compared to the control group (Fig. 7C). However, the expression of PSD95 was significantly increased only in the group treated with Hy7714-derived exosomes (*p* = 0.0009). The reduced synaptic plasticity-related protein expression in control group is consistent with previous findings that Aβ oligomers reduce the strength and plasticity of glutamatergic synaptic transmission [51, 52]. A number of studies have reported that the treatment with metabolites demonstrates neuroprotective effects and improves memory functions through epigenetic mechanisms. This is achieved by upregulating the expression of genes encoding neurotrophic factors [53, 54]. Furthermore, while GMEVs do not directly cause or treat disease, they can indirectly influence diseases by transmitting both harmful and beneficial effects. Gut probiotics frequently exhibited positive neurological effects and some of these effects could potentially be replicated by GMEVs [55, 56]. Indeed, the previous studies have demonstrated that in specific diseases, probiotics can exert their effects through small molecule substances, such as microRNAs and lipids, which are encapsulated in GMEVs [57, 58]. Our results demonstrate that treatment with microbe-derived metabolites and exosomes enhances axonal growth and synaptic activity as compared to the control group in Alzheimer’s disease models. Based on these results, we suggest that microbe-derived metabolites secrete or modulate neurotrophic factors. The exosomes deliver exogenous RNA cargo, which may contribute to the alleviation of neuroinflammatory diseases. Therefore, we confirmed the potential of microbe-derived metabolites and exosomes as therapeutic candidates for the treatment of Alzheimer’s disease (Fig. 7).

## Conclusion

We demonstrate that microbe-derived metabolites and exosome exposure during neural development may induce neural differentiation, promote synapse-related protein expression in human iNSCs-derived neurons in a gut-brain axis chip. Furthermore, we observed that these microbe-derived metabolites and exosomes may have protection effect on neural cells in Alzheimer's disease. Therefore, the gut-brain axis chip system can serve as a powerful culture platform for studying the interaction of microbial-derived metabolites and exosomes. However, the further research is needed to address gaps in our understanding of the impact of microbial-derived metabolites and exosomes on neurodevelopment and neurodegenerative diseases.

## Data Availability

The authors have no data to share since all data are shown in the manuscript.

## Abbreviations

iPSCs: Induced pluripotent stem cells
iNSCs: Induced neural stem cells
Caco-2: Human intestinal epithelial cells
PDMS: Polydimethylsiloxane
EVs: Extracellular vesicles
GMEVs: Gut microbiota-derived EVs
CNS: Central nervous system
GABA: Gamma-aminobutyric acid
SCFAs: Short-chain fatty acids
BDNF: Brain-derived neutrotrophic factor
NGF: Nerve growth factor
GDNF: Glial cell line-derived neurotrophic factor
